# Identification of multiple independent horizontal gene transfers into poxviruses using a comparative genomics approach

**DOI:** 10.1186/1471-2148-8-67

**Published:** 2008-02-27

**Authors:** Kirsten A Bratke, Aoife McLysaght

**Affiliations:** 1 Smurfit Institute of Genetics, University of Dublin, Trinity College, Dublin 2, Ireland

## Abstract

**Background:**

Poxviruses are important pathogens of humans, livestock and wild animals. These large dsDNA viruses have a set of core orthologs whose gene order is extremely well conserved throughout poxvirus genera. They also contain many genes with sequence and functional similarity to host genes which were probably acquired by horizontal gene transfer.

Although phylogenetic trees can indicate the occurrence of horizontal gene transfer and even uncover multiple events, their use may be hampered by uncertainties in both the topology and the rooting of the tree. We propose to use synteny conservation around the horizontally transferred gene (HTgene) to distinguish between single and multiple events.

**Results:**

Here we devise a method that incorporates comparative genomic information into the investigation of horizontal gene transfer, and we apply this method to poxvirus genomes. We examined the synteny conservation around twenty four pox genes that we identified, or which were reported in the literature, as candidate HTgenes. We found support for multiple independent transfers into poxviruses for five HTgenes. Three of these genes are known to be important for the survival of the virus in or out of the host cell and one of them increases susceptibility to some antiviral drugs.

**Conclusion:**

In related genomes conserved synteny information can provide convincing evidence for multiple independent horizontal gene transfer events even in the absence of a robust phylogenetic tree for the HTgene.

## Background

Horizontal gene transfer (HGT, also: lateral gene transfer) is an important process in viral evolution. HGT describes an event that differs from the regular 'vertical' transfer of genes from parent to offspring: for example, host gene capture in viruses, where pieces of host DNA are incorporated into the newly formed virion. HGT is thought to play a major role in evolution, as there has apparently been frequent transfer of protein domains or entire genes within and between all divisions of life with important implications for molecular phylogeny studies [1-3]. Especially in pathogens these horizontally transferred genes (HTgenes) are of enormous interest as they often increase virulence by encoding homologs to the host's immune system and interfering with it [4-6]. Multiple independent horizontal gene transfer events are thus particularly significant; they highlight the advantage that is conferred on the virus genome by a gene that has been gained and maintained several times.

Poxviruses are large double-stranded DNA viruses (up to 360 kb) that infect a wide range of hosts from insects to mammals. They are classified into entomopox (EPV, insect-infecting) and chordopox viruses (ChPV, vertebrate-infecting), with ChPV sub-divided into at least nine genera. Several members of the *Poxviridae *are associated with human disease, including variola (smallpox), *Molluscum contagiosum *and monkeypox, which has been classifed as an emerging virus [7,8] and was responsible for an outbreak of monkeypox in the US in 2003. The most well known poxviruses are smallpox, an orthopox virus (OPV) responsible for devastating human pandemics until it was declared eradicated by the WHO in 1980, and cowpox, used in the development of the first vaccine by Edward Jenner in 1796. Over 50 genomes have been fully sequenced and many attempts have been made to reconstruct a robust phylogeny of poxviruses [9-12].

Poxviruses remain in the cytoplasm of the host cell and to a large extent provide their own replication machinery. Many of these genes are not part of a viral core set and it is possible that they were acquired from an ancient host cell by a poxvirus ancestor [13]. The fact that poxviruses do not enter the nucleus poses a practical problem when it comes to the mechanism of host gene capture: how HGT from host to virus takes place is not yet known, but as poxvirus genes lack introns, HGT may proceed through reverse transcription of host mRNAs and integration of the cDNA into the virus genome. [14]. It has recently been suggested that certain poxviruses may act as vectors for the transfer of transposable elements between animal phyla [15].

There are several approaches to detecting HGT, all of which are best used in conjunction with each other [16]. The first evidence usually comes from sequence similarity to a gene in a distantly related organism or in the viral host. Phylogenetic gene trees which include sequences from both viruses and cellular organisms can also provide an indication of host gene capture where virus genes group closely with homologs from the host or organisms related to the host, i.e. where gene history differs from species history [17]. Atypical base composition of a particular gene, for example a GC content that differs significantly from that of most other genes in the genome, provides another piece of evidence that HGT may have recently taken place and this approach has been used to detect host-derived regions in poxviruses [18] and other small genomes [19]. This signal, however, is expected to fade over time and therefore this approach is only useful for detecting recent HGT.

These difficulties are compounded when one wishes to test whether there have been multiple independent HGT events of the same gene into related viruses. This may be indicated by a polyphyletic relationship in the phylogenetic tree, but such a tree would require strong support (e.g., high bootstrap values) for several branches in order to be reliable – frequently this support is not there. A mixed pattern of presence and absence of a particular gene in a group of closely related organisms is another indication for horizontal transfer of the gene in question, but an ancestral origin with loss in some genomes can also explain this observation.

We use comparative gene order information to distinguish between a shared history of the HTgene in the virus genomes and several independent transfer events. Where a given HTgene is in an equivalent location in terms of its neighboring genes in all of the genomes only a single transfer event is supported. A HTgene that is located in a different genomic region in related viruses strengthens the case for several transfer events rather than a common origin. This is an especially strong tool in poxviruses where the gene order is highly conserved throughout the syntenic core of the genome [9,20].

We systematically searched for evidence of HGT between poxviruses and other organisms using strict search criteria and identified nine candidate HTgenes, some of which had been previously reported. We examined both the tree topology and the genomic context for evidence of the number of HGT events. We also applied the comparative synteny method to several candidate horizontally transferred genes reported in the literature [11,21].

## Results and Discussion

### Poxvirus gene families and species phylogeny

Poxvirus genes were grouped into families based on protein sequence similarity (see Methods). 14,936 genes were grouped into 730 families of a least three members. Of these, 33 gene families were found in single copy in each of the completely sequenced genomes; we labelled these "core families" for our purposes (Table 1). A further 523 gene families were found in single copy in a subset of the completely sequenced genomes; we labelled these "ortholog families". Finally, 174 families were defined that contained both orthologs and paralogs; we labelled these "ortho-paralog families".

**Table 1:** Core Conserved Protein Families

Vaccinia Gene	Putative Function
A2L	Transcription factor VLTF
A3L	Viral core protein p4b
A7L	Subunit of VETF (82 kDa)
A10L	Virion core protein p4a
A11R	Virion formation
A16L	Myristylated protein/membrane protein
A18R	DNA helicase/transcriptional elongation factor
A22R	Holliday junction endonuclease/palmitylprotein
A23R	Subunit of VITF-3 (45 kDa)
A24R	RNA polymerase subunit rpol 32
A32L	ATPase/DNA packaging/virion assembly
D5R	NTPase
D6R	Subunit of VETF (70 kDa)
D11L	ATPase/nucleoside triphosphate phosphohydrolase/transcription termination factor
D12L	Subunit of mRNA capping enzyme/transcription termination factor
E1L	Poly A polymerase subunit
E6R	Hypothetical protein
E9L	DNA polymerase
E10R	Sulfhydryl oxidase/redox protein/S-S bond formation/virus assembly
F9L	Membrane protein/S-S bond formation pathway protein
F10L	Serine/threonine kinase
G5R	Ribonucleotide reductase/morphogenesis
G7L	Structural virion core protein
G9R	Myristylated protein/membrane protein
H2R	Late protein
H6R	DNA topoisomerase
I7L	Virion core cysteine protease
I8R	RNA helicase
J3R	Poly A polymerase subunite/cap methyltransferase
J5L	Membrane protein
J6R	RNA polymerase subunit
L1R	ImV envelope/membrane protein
L3L	Hypothetical protein

**Figure 1:**
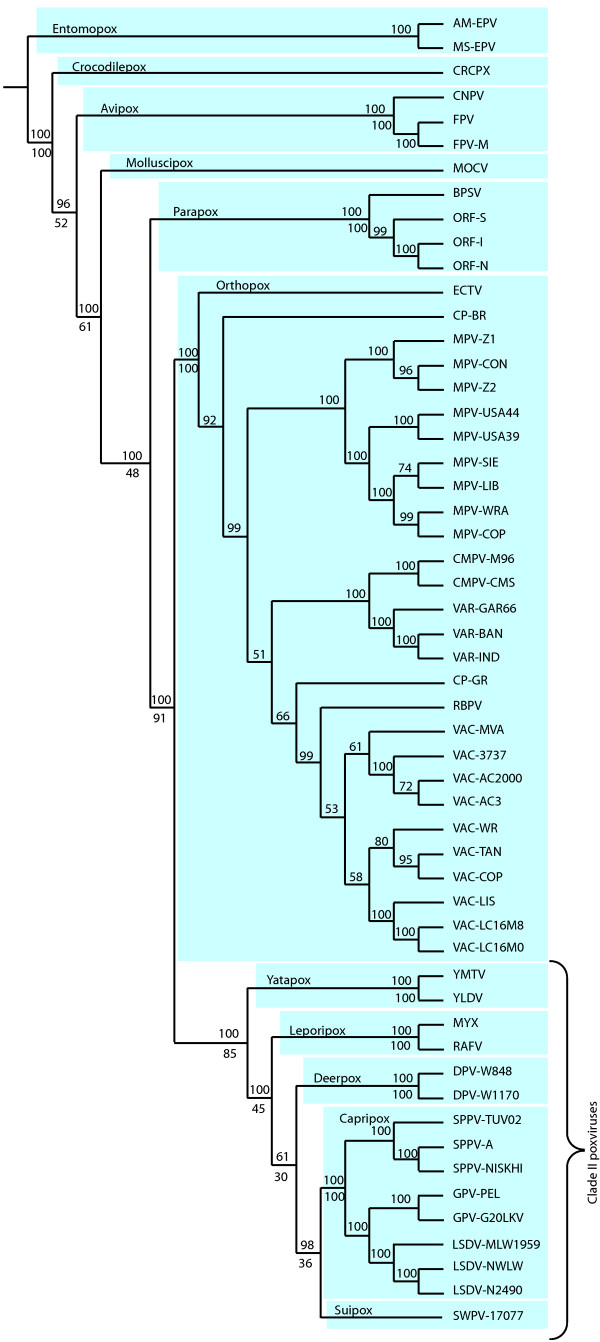
**A schematic representation of the ML species tree topology**. Branch lengths are not to scale. Numbers above branches are percent bootstrap values, numbers below the branches indicate the percentage of gene trees that supported the branch. Taxonomic groups are labelled and shaded for clarity. The term "clade II" poxviruses is used for simplicity and refers to the group of yatapox, deerpox, capripox and suipox, as per convention [11].

We used concatenated amino acid (aa) sequence alignments of the core families to estimate the poxvirus phylogeny under different reconstruction methods. The maximum-likelihood (ML) tree of poxviruses (Figure 1) incorporates 54 complete genomes (Table 2) and agrees with previously reported phylogenies in terms of the relative positions of genera [9-11]. The ML and neighbor-joining (NJ) trees were identical except for NJ grouping parapox and molluscipox as sister clades with high bootstrap support. The maximum-parsimony (MP) tree agreed with this grouping of parapox and molluscipox and also supports capripox as the outgroup of suipox and deerpox, rather than suipox and capripox being monophyletic as suggested by the ML topology. Additionally, we examined the congruence of individual gene trees with the species tree topology depicted in Fig. 1. In most cases the gene trees were in perfect agreement with the inferred species tree, lending further support to the topology.

**Table 2:** Complete Poxvirus Genomes

Code	Full Name	GenBank Accession	Genus
AM-EPV	Amsacta moorei entomopoxvirus	AF250284	Entomopox
MS-EPV	Melanoplus sanguinipes entomopoxvirus	AF063866	Entomopox
CRCPX	Crocodilepox virus	DQ356948	†
CNPV	Canarypox virus strain ATCC VR-111	AY318871	Avipox
FPV-I	Fowlpox virus	AF198100	Avipox
FPV-M	Fowlpox virus isolate HP-438 (Munich)	AJ581527	Avipox
MOCV	Molluscum contagiosum virus subtype 1	U60315	Molluscipox
BPSV	Bovine popular stomatitis virus strain BV-AR02	AY386265	Parapox
ORF-S	Orf virus strain OV-SA00	AY386264	Parapox
ORF-I	Orf virus strain OV-IA82	AY386263	Parapox
ORF-N	Orf virus strain NZ2	DQ184476	Parapox
ECTV	Ectromelia virus strain Moscow	AF012825	Orthopox
CP-BR	Cowpox virus strain Brighton Red	AF482758	Orthopox
MPV-Z1	Monkeypox virus strain Zaire-96-I-16	AF380138	Orthopox
MPV-CON	Monkeypox virus strain Congo_2003_358	DQ011154	Orthopox
MPV-Z2	Monkeypox virus strain Zaire_1979-005	DQ011155	Orthopox
MPV-USA44	Monkeypox virus strain USA_2003_044	DQ011153	Orthopox
MPV-USA39	Monkeypox virus strain USA_2003_039	DQ011157	Orthopox
MPV-SIE	Monkeypox virus isolate Sierra Leone	AY741551	Orthopox
MPV-LIB	Monkeypox virus strain Liberia_1970_184	DQ011156	Orthopox
MPV-WRA	Monkeypox virus strain WRAIR7-61	AY603973	Orthopox
MPV-COP	Monkeypox virus strain COP-58	AY753185	Orthopox
MPV-M96	Camelpox virus M-96 (Kazakhstan)	AF438165	Orthopox
CMPV-CMS	Camelpox virus CMS	AY009089	Orthopox
VAR-GAR66	Variolar minor virus strain Garcia-1966	Y16780	Orthopox
VAR-BAN	Variola major virus strain Bangladesh-1975	L22579	Orthopox
VAR-IN	Variola virus strain India-1967	X69198	Orthopox
CP-GR	Cowpox virus strain GRI-90	X94355	Orthopox
RBPV	Rabbitpox virus	AY484669	Orthopox
VAC-MVA	Vaccinia virus strain Acambis 3000 Mod. Virus Ankara	AY603355	Orthopox
VAC-3737	Vaccinia virus strain 3737	DQ377945	Orthopox
VAC-AC2000	Vaccinia virus strain Acambis clone 2000	AY313847	Orthopox
VAC-AC3	Vaccinia virus strain Acambis clone 3	AY313848	Orthopox
VAC-WR	Vaccinia virus Western Reserve	AY243312	Orthopox
VAC-TAN	Vaccinia virus strain Tian Tan	AF095689	Orthopox
VAC-COP	Vaccinia virus Copenhagen	M35027	Orthopox
VAC-LIS	Vaccinia virus strain Lister	AY678276 (*)	Orthopox
VAC-LC16M8	Vaccinia virus strain LC16m8	AY678275	Orthopox
VAC-LC16M0	Vaccinia virus strain LC16mO	AY678277	Orthopox
YMTV	Yaba monkey tumor virus	AY386371	Yatapox
YLDV	Yaba-like disease virus	AJ293568	Yatapox
MYX	Myxoma virus strain Lausanne	AF170726	Leporipox
RAFV	Rabbit fibroma virus	AF170722	Leporipox
DPV-W848	Deerpox virus W-848-83	AY689436	†
DPV-W1170	Deerpox virus W-1170-84	AY689437	†
SPPV-TUV02	Sheeppox virus 10700-99 strain TU-V02127	AY077832 (*)	Capripox
SPPV-A	Sheeppox virus strain	AY077833 (*)	Capripox
SPPV-NISKHI	Sheeppox virus strain NISKHI	AY077834 (*)	Capripox
GPV-PEL	Goatpox virus strain Pellor	AY077835 (*)	Capripox
GPV-G20LKV	Goatpox virus strain G20-LKV 19	AY077836 (*)	Capripox
LSDV-NLW1959	Lumpy skin disease virus isolate Neethling vaccine LW1959	AF409138	Capripox
LSDV-NWLW	Lumpy skin disease virus isolate Neethling Warmbaths LW	AF409137	Capripox
LSDV-N2490	Lumpy skin disease virus isolate Neethling 2490	AF325528	Capripox

* Genome was not annotated at time of analysis and genes were predicted using the EMBOSS getorf algorithm.

Within-genus relationships for genera with many representatives were inferred by the inclusion of as many gene sequences as possible. For parapox, capripox and orthopox, we aligned all ortholog families conserved in the genus of interest (i.e. genes connected by yellow lines in Additional File 1) and inferred trees for those genera using ML and NJ (see Methods). NJ and ML results were congruent and the bootstrapped ML results are shown in Fig. 1.

### Evidence for horizontal gene transfer from sequence similarity

We identified 14 pox gene families that included at least one member with a significant PSI-BLAST hit to a gene in a non-pox organism (see Methods for search criteria). The search protocol we employed is conservative and we did not detect some previously reported non-pox homologs of poxvirus proteins (Table 3; lower section and refs [11,21]). However, we initially wished to confine our gene order comparisons to genes with convincing amino acid sequence similarity to genes found outside poxviruses. Of these 14 gene families, some did not warrant further investigation. The *gag*, *pol *and *env *open reading frames found in avipox are part of a reticuloendotheliosis virus (REV) sequence that is inserted into the pox genome and groups with sequences from REV and other avian viruses. While this is HGT of sorts, it is a special case where the REV sequence can reform into infectious particles after poxvirus infection of a chicken host [22] and these genes were not investigated further here. The fusolin/enhancing factor (EF) family was found to be present only in incompletely sequenced entomopox genomes and one insect larva genome (*Pseudaletia separata*). The similarities between the EF and fusolin genes of different EPV and their detrimental effect on the host's resistance to the viruses have been reported [23], but it appears there has been an annotation mistake in the record of the insect sequence [GenBank: JC5185], as the paper it refers to [23] makes no mention of an insect sequence, and it is identical to that of the insect's EPV [GenBank: BAA09138]. As we are unsure of the veracity of the candidate host sequence, we excluded this gene from further analysis. Ubiquitin is also found by our search. The large number of homologs makes further investigation difficult, and as this protein is shared by all eukaryotes, it is likely to be ancestral to all poxviruses. The remaining 9 protein families were studied in more detail and the HGT histories suggested by our analyses are summarised in Table 3.

**Table 3:** Gene Families With Potential HGT

			*Number of HGT events*		
Gene Family	Poxvirus Genera	**Family Size **¶	Phylogeny	Gene Order	Reference
*Candidate HTgenes identified by PSI-BLAST search*					

dUTPase	entomo, avi, para, ortho, clade II	137	2 †	*	[51,52]
Glutathione peroxidase	avi, mollusci	42	2 §	2	[11]
GNS1	avi	18	1	1	
Interleukin-10	avi, para, yata, capri	145	2 †§	≥ 2	[11,25]
Ribonucleotide reductase, large subunit	ortho, sui	90	1	1	[11]
Ribonucleotide reductase, small subunit	croc, avi, or-tho, clade II	178	3 †	2	[11,37]
S1R/CGI-119	ortho	36	1	1 ‡	
Serpin	avi, ortho, clade II	272	1 §	-	[11,53]
Thymidine kinase	entomo, avi, ortho, clade II	143	? †§	2–3	[29,34]

*Candidate HTgenes from the literature*					

NlpC/P60 peptidase (G6R)	all			1	[21]
CD47	ortho, clade II			1	[11]
Deoxycytidine kinase	avi			1	[11]
Glutaredoxin	ortho			1	[11]
Hydroxysteroid dehydro-genase	avi, mollusci, ortho, yata, deer, sui			1	[11]
MHC 1	yata, sui, deer			1	[11]
Deoxyribodipyrimidine photolyase	entomo, avi, lepori			≥ 2	[11]
α(2,3)-sialtransferase	lepori, deer			1	[11]
α-soluble NSF attachment protein (SNAP)	avi			1	[11]
Profilin	ortho			1	[11]
DNA-directed RNA poly-merase, subunit A	all			1	[11]
Carbonic anhydrase	ortho, lepori			1	[11]
Superoxide dismutase	entomo, ortho, lepori, sui, deer, capri			1	[11]
Uracil DNA glycolase	entomo			*	[11]
Interferon gamma receptor	ortho, lepori, sui, deer, capri			*	[11]

¶ Poxvirus family members and all homologs identified in PSI-BLAST search

† No bootstrap support (≥ 80%) for critical branches

‡ Followed by gene duplication

§ Previously described as polyphyletic in poxviruses

* Insufficient synteny conservation

### Evidence from gene order comparisons

Poxviruses have extremely well conserved gene order, particularly of the core families (Additional File 1 and [9]). We estimated the overall level of synteny conservation throughout poxvirus genomes by considering the conservation of neighboring genes between orthologs in pox. For these purposes we examined only genes and neighbors which had been grouped into families without paralogs and which were present in at least two complete genomes. We considered that if a given gene always has the same two immediate neighbors in every genome in which it is found, then it has perfect neighbor conservation. By contrast, if the neighbors are always different, then there is zero conservation. We measured this using the following statistic: (M-N)/(M-2); where M is the maximum possible number of unique neighbours (i.e., different neighbors in every case) and N is the observed number of neighbours. This will always return a value between 0 and 1. We found that 69% of ortholog families had a neighbor conservation value of at least 0.9 and that 89% had at least 0.7 neighbor conservation.

**Figure 2:**
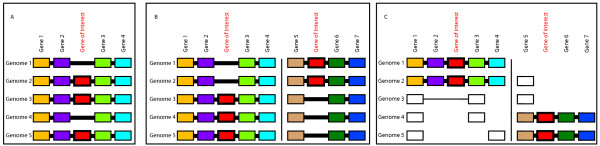
**Model gene order comparisons for inferring the number of horizontal gene transfer events**. The relative order of genes around the gene of interest is illustrated. Orthologous genes are shaded in the same color and lined up vertically. A white box indicates that the gene is present in the genome, but not at the expected (i.e., equivalent) location. Horizontal lines indicate genomic segments. Thick horizontal lines indicate that the connected genes are neighboring genes of the specified type (where gene of interest is present) or have fewer than three intervening genes of the specified type (where gene of interest is absent). Thin horizontal lines signify three to six intervening genes. Vertical lines separate discontiguous genomic regions. (A) The gene of interest is in an equivalent location in all genomes where it is found. This supports a single origin (i.e., horizontal transfer) of this gene. (B) The gene of interest is found in two different genomic locations and the relative arrangement of other genes in each of these locations is conserved (i.e., there is no support for a local rearrangement). This supports two independent transfers of this gene. (C) The gene of interest is found in two different genomic locations but these regions are not conserved between genomes, so it is not possible to infer if it was a single transfer followed by genome rearrangement, or two independent transfer events.

This conservation permits the comparison of the genomic location of HTgenes between genomes. Shared genomic context may also be useful for distinguishing orthologs and paralogs in conjunction with phylogenetic trees, though that analysis is not conducted here. These comparisons of HTgene locations are done with respect to gene families which are shared between the genomes of interest. Depending on the extent of gene order conservation around the HTgene between genomes it may be possible to distinguish single from multiple transfer events (Fig. 2A and 2B). If the local gene order is not well conserved then a single transfer followed by genome rearrangement cannot be excluded even when the HTgene is present in a completely different context in different genomes (Fig. 2C). Within poxvirus genera this is a particular issue when comparing EPV gene order to that of ChPV, and to a certain degree also in comparison of avipox to other ChPV because of the high amount of rearrangement (Additional File 1).

We made three kinds of illustrations of the genomic context of HTgenes, distinguished by the type of gene families that the HTgene is plotted alongside. In the first kind the HTgene location is plotted with respect to the "core families" only. There are only 33 of these genes, so the distances within the diagram may be quite large, however because these are shared by all pox genomes, this view permits a low-resolution comparison across all genera. The second type of diagram plots the HTgene with respect to ortholog families, which are much greater in number. This increases the "resolution" of the plot and still avoids the confounding effect of paralogous genes. Finally we plot the location of the HTgene with respect to any of the gene families and examine it with the caveat that paralogous genes may be misleading.

We applied this analysis to the nine HTgenes uncovered by our PSI-BLAST search and compared the results to the scenarios supported by ML and NJ tree of the pox gene family and the non-pox homologs. We also applied the comparative gene arrangement analysis to candidate HTgenes from the literature (Table 3).

In the case of dUTPase and Uracil DNA glycolase there was insufficient gene order conservation to allow comparisons – this reflects the fact that many of the transfers were into the poxvirus chromosome ends where the genome appears to be more labile. Also, the serpin gene family contains many paralogs within poxviruses which made comparison of gene order between viruses intractable. However, we found that in most cases gene order comparisons between the poxviruses can distinguish between single or multiple origins of a HTgene in poxviruses, even in the absence of a robust phylogenetic tree. The gene location comparison provided supporting evidence for at least two independent origins of: Glutathione Peroxidase; Interleukin-10; Ribonucleotide Reductase, small subunit; Thymidine Kinase; Deoxyribo-Dipyrimidine Photolyase (discussed below). In some cases, such as Interleukin-10, the synteny information is suggestive of more HGT events than were apparent from the phylogenetic tree alone. In other cases, such as Ribonucleotide Reductase, small subunit, the converse was true, and the synteny data indicated that there were fewer events than did the (poorly supported) phylogenetic tree.

### Interleukin-10

Interleukin-10 (IL-10) is a multifunctional cytokine that suppresses inflammation, antiviral response and other host functions. It inhibits activation and maturation of dendritic cells and thus reduces their antigen-presenting ability and inhibits cytokine synthesis via T- and NK- cells. Homologs of IL-10 have been found in many different viruses [24-26]. Viral IL-10 has been shown to play an important role in delaying the development of acquired immunity to orf virus in humans [27] and to vaccinia in mice [28]. The origin of some viral homologs to IL-10 has been investigated and it is suggested that orf virus (a parapoxvirus) captured the gene from sheep or goat [25,29].

The NJ tree for IL-10 genes (based on their aa sequences) found in pox and other organisms indicates that there are two origins of this gene in poxviruses because the pox genes group into two clades, i.e., they are polyphyletic (Additional File 2; and [11]). However, the low bootstrap support for the tree topology means that this tree alone does not give strong support for multiple horizontal transfer events. In fact, the ML tree has a different topology indicating monophyly and also has low bootstraps (Additional File 2). In this case, the lack of clarity in the phylogenetic tree possibly stems from a lack of information within the alignment (Additional File 3), as indicated by the low bootstrap support.

**Figure 3:**
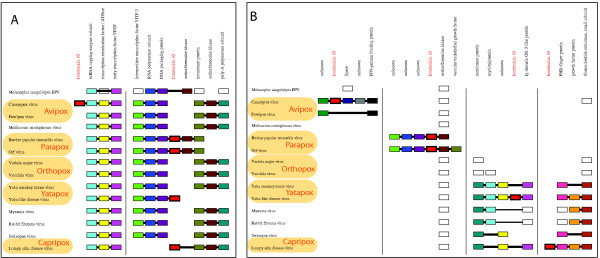
**Synteny conservation around IL-10 in poxvirus genomes**. As for Fig. 2. The gene of interest, IL-10, is colored red with a bold outline. A horizontal line that crosses a box, indicates that this gene is not part of the contiguous segment. (A) Neighborhood with respect to "core families". The Serine/Threonine Kinase gene has experienced a local inversion in the parapox genomes and is shown in two columns for clarity; (B) Neighborhood with respect to "ortholog families".

The synteny diagram with respect to core families (Fig. 3A) suggests that the canarypox IL-10 is not at the expected location, however, these data cannot exclude the possibility of genome rearrangement altering the location of the canarypox IL-10 gene because the environment observed in orthopox is broken by rearrangements in avipox.

Including more genes in the synteny comparison distinguishes the genomic locations of the yatapox and capripox IL-10 genes as each is present in a region of genome that is well-conserved between these pox genera (thus permitting comparison) yet different for the two sets of viruses (Fig. 3B), indicating distinct origins for the yatapox and capripox IL-10 genes. This same distinction was found by Hughes based on phylogenetic analysis [11]. The IL-10 genomic locations in parapox and avipox are not sufficiently conserved to permit comparison. However, we note that if one or both of these originated in the same event as the yatapox or capripox IL-10 then that also requires many loss events in other pox genera as well as a loss and subsequent re-acquisition in either yatapox or capripox. The gene order with respect to all neighboring gene families (figure not shown) supports the distinction of yatapox and capripox IL-10 but is not otherwise informative of the history of IL-10 due to lack of significant conservation, so cannot distinguish these possibilities. We therefore infer at least two independent HGT events of the IL-10 gene from vertebrates to poxviruses.

### Thymidine Kinase

Thymidine kinase (TK) is present in many poxviruses [30-32]. It phosphorylates a wide range of substrates and, surprisingly, has been found to increase susceptibility to antiviral drugs by selectively phosphorylating some thymidine analog drugs to a greater degree than the host TK1 gene and thus increasing the efficacy of the antiviral drug [33]. Separate origins for avipox and orthopox TK have been suggested based on phylogenetic evidence [29,34] and this is supported here by the different genomic environments in avipox and other chordopox (Additional File 4; and [35]).

The gene order is completely scrambled in entomopox, however, so these data do not distinguish between shared origins for entomopox and either the avipox or other chordopox TK genes and distinct origins (i.e., the data do not distinguish between two or three transfer events). The former scenario would require that the TK gene was present in the common ancestor of all pox and subsequently lost in either avipox or the other chordopox only to be regained independently. If the entomopox and avipox TK gene are descended from the same HGT event, then this scenario would require three events (HGT into pox ancestor, loss from chordopox excluding avipox, HGT into another chordopox lineage). In a simplistic sense this is equally parsimonious with three HGT events. A more biologically realistic analysis should include consideration of any selection to retain, lose or acquire this gene; properties that we do not currently know. The fact that this gene has been acquired and retained independently at least twice indicates that multiple HGT events are possible. Loss and reacquisition in the chordopox lineage may indicate significant changes in the environment have changed the nature of the selection acting on this gene.

In avipox, the TK gene and the adjacent HT-motif protein gene (HT) are in the location occupied by ribonucleotide reductase, large subunit (RRL) in orthopox and suipox. This, along with reported 15 bp direct repeats flanking TK and HT led to the suggestion that these genes transposed within avipox to this location in avipox and replaced RRL [36]. This suggestion is at odds with the experimental strategy employed here because if intra-genomic translocations are a common occurrence it would mean that completely distinct genomic neighborhoods are not sufficient to indicate distinct origins. However, there is no real support for this transposition hypothesis from the many data now available: (i) The phylogenetic evidence supports an independent origin of avipox TK; (ii) There is no evidence that the RRL gene was ever present in avipox because it is not found outside orthopox and suipox (whose most recent common ancestor is not shared with avipox); (iii) Furthermore, we cannot identify the 15 bp repeat referred to in the 1992 paper [36] but we did identify a 12 bp direct repeat (starting nucleotide positions 87698 and 88647 in AF198100). One of the repeat copies is inside the open reading frame of the I5L gene, which adds the constraint that if it is the result of an insertion-site duplication then it must not have disrupted the function of this gene. The relationship of the entomopox TK gene to the other pox TK genes remains inconclusive, with a third independent transfer being one possibility.

We conclude from the gene order data that there were at least two independent horizontal transfers of TK into poxviruses, and possibly a third (into entomopox). The independent transfer into the same locus of the TK gene in avipox and the RRL gene into the ancestor of orthopox and suipox suggests the intriguing possibility that this genomic location has properties that make it particularly receptive to gene transfers.

### Ribonucleotide Reductase, small subunit

Ribonucleotide reductase (RR) functions as a heterodimeric tetramer and leads to the reduction of all four ribonucleotides to deoxyribonucleotides in DNA biosynthesis. The small subunit (RRS) was sequenced in vaccinia and found to be highly similar to eukaryotic RSS genes, including the mouse RSS, suggesting host gene capture [37]. RRS is present in most chordopox genera except parapox, molluscipox and some avipox. The avipox RRS gene is present in a different synteny block than the RRS gene in other chordopox (Additional File 5). These data support two independent transfers of RRS into pox: one into the avipox lineage, and one into the ancestor of orthopox and clade II.

### Glutathione Peroxidase

The viral glutathione peroxidase (GP) protects virus and infected cells against oxidative damage resulting from interaction with the immune system [38]. It is not present in orthopox and was first identified in molluscum contagiosum virus [39]. GP is found in avipox and molluscipox and separate transfer events have previously been suggested [11]. The synteny diagram with ortholog families clearly shows that the gene is in an equivalent location in all avipox genomes (Additional File 6), but is in a distinct location in molluscipox and thus supports independent transfers of this gene into these two lineages.

### Deoxyribodipyrimidine Photolyase

Photolyases are enzymes which catalyse the repair of UV-induced DNA damage. In poxviruses, photolyases are implicated in the survival of the virus outside host cells in between infections [40]. Deoxyribodipyrimidine Photolyase (DP) is found in entomopox, avipox and leporipox. It was identified as a candidate HTgene through sequence similarity with genes in insect and vertebrate genomes [11]. The avipox gene lies in a region of conserved synteny with other ChPV, but the DP gene is not at the syntenous location in leporipox (Additional File 7). This supports independent origins for the avipox and leporipox DP genes. It is not possible to compare the location of the entomopox genes with each other or with ChPV due to insufficient synteny conservation. We conclude that there were at least two independent transfers of DP into poxviruses.

## Conclusion

Here we have gone beyond the comparison of the sequences of homologous genes and have introduced comparative synteny data into the analysis of horizontal gene transfer. These data are completely independent of phylogenetic tree and sequence composition data. For the 24 genes with evidence of horizontal transfer analysed here, all but four had sufficiently conserved synteny between poxviral genomes to permit comparison. In five cases, the presence of the HTgene in a well-conserved, yet distinct, region in different poxvirus genomes supported a conclusion of multiple independent transfers of a homologous gene into *Poxviridae*. In the other cases, this study confirmed that a single event gave rise to the HTgene in poxviruses.

This strategy is based on the assumption that there is no bias in the insertion site of a HTgene with respect to the neighboring genes in the receiving (pox) genome. The only known bias in terms of the location of new genes in poxviruses is that they tend to be located towards the ends of chromosomes [9] and this is not expected to influence the neighbors for this analysis. More importantly, we interpret the conservation of synteny across poxvirus genomes as evidence against genomic rearrangement events in that region. This means that under these circumstances we can exclude the possibility that the new genomic location is caused by genome rearrangement within the pox lineage rather than independent transfer into the lineage.

A major strength of this method is that it does not rely on phylogenetic trees (and thus multiple sequence alignments) in order to interpret the number of transfer events. In previous studies many HTgenes were refractory to phylogenetic analysis due to difficulties rooting the trees or insignificant support for critical branches (e.g. [11] in pox, and [41]). Phylogenetic trees are frequently plagued by problems which abolish their usefulness for analysis of horizontal gene transfer [42]. Because the conserved synteny data are independent of the phylogenetic tree for the HTgene these results could be combined.

The HTgenes with multiple origins identified here have very interesting biological properties. The presence of viral TK increases susceptibilty to antiviral drugs, a surprising finding [33]. The particular benefit to the virus, if any, of having this gene is not clear. Three other HTgenes, IL-10, GP and DP are known to improve the survival of the virus against the host immune system (IL-10) and environmental damage (GP and DP). In the case of these genes it is easy to speculate that they are particularly advantageous to the virus, and the fact that they have been successfully transferred into poxvirus genomes on more than one occasion lends support to this hypothesis.

## Methods

### Family Definitions

We downloaded all available poxvirus proteins from GenBank in June 2006 and predicted proteins for unannotated complete genomes using the EMBOSS *getorf *application [43] (total 17,185 sequences). These proteins were compared in an all-against-all BLAST search using default parameters [44]. We used The Markov Cluster Algorithm (MCL) [45] to group proteins into families with the Centering parameter set to 1.2 and the Inflation parameter set to 1.2. These parameters were chosen after experimentation in order to maximize the family size. Alignments of all of these families were performed using MUSCLE and are provided as supplementary material (Additional File 8).

### Species Phylogeny Construction

Thirty-three families were found in single-copy in every completely sequenced poxvirus genome ("core families"; Table 1). We generated alignments of the protein sequences of each of these genes using MUSCLE [46] and concatenated the alignments. Based on this concatenated sequence alignment, phylogenetic trees of 54 completely sequenced poxviruses (Table 2) were reconstructed by different methods: NJ (PHYLIP package [47] and ClustalW [48]), MP (PHYLIP package) and ML (PhyML package [49]). The best models for ML analysis were estimated using PhyML [49] by experimenting with a range of settings for several parameters (gamma parameter, substitution model, number of substitution rate categories) and choosing the ones that gave the highest likelihood. The JTT and TN93 models were used for protein and nucleotide alignments, respectively. To allow for bootstrapping, a less computationally intensive, representative phylogeny including at most two members of each genus was constructed. Single gene trees were also constructed using PhyML from the 33 conserved families, and their congruency with the concatenated gene tree was noted. To infer within-genus relationships, ortholog families shared by all members of a genus were aligned at the protein level and the gaps were copied into the nucleotide sequence to generate nucleotide (nt) alignments. The nt alignments were concatenated and the tree of the genus was inferred using PhyML with the TN93 model. For comparison we also constructed the trees using NJ (with both the PHYLIP package and ClustalW).

### Detection of non-poxviral homologs

We used PSI-BLAST [50] to search all 17,815 poxvirus proteins against the GenBank non-redundant (nr) database (May 2007; 4.9 million sequences). The e-value threshold for inclusion in the PSSM was e-20, with a maximum of five iterations. Only alignments of 70% identity or higher and at least 80% length of query sequence were kept as homologs. We selected these parameters after some experimentation with different values in order to retrieve only strong candidates for HGT and to keep detection of false positives to a minimum. We excluded hits to synthetic constructs (e.g., artificial vectors).

### Gene Phylogenies

The protein sequences of members of the nine gene families with PSI-BLAST hits outside poxviruses were aligned with their non-poxviral homologs using MUSCLE [46]. NJ trees were drawn with ClustalW [48] and bootstrapped 1000 times. ML trees were drawn with PhyML using model JTT, proportion of invariable sites = 0, number of substitution rate categories = 6 and estimating the gamma distribution parameter.

### Synteny Diagrams

Three kinds of comparative genomic plots were produced. Only genes that have been classified into families are considered. (1) A low resolution plot illustrates the location of the gene of interest in each genome with respect to "core families". The actual distance between genes on this plot may be quite large. (2) A medium resolution compares the location of the HTgene with respect to "ortholog families". (3) The maximum resolution plot includes neighboring genes from any families.

In all of these diagrams when the gene of interest is present in a given genome, the genes plotted along side are immediate neighbors (of the given type) with no intervening genes. That is, in the ortholog families plot, the genes plotted are all the nearest-neighbor genes with identified orthologs in any genome.

When the gene of interest is absent from a given genome, we allowed some intervening genes and indicate this with the thickness of the line (as per figure legend). We chose ranges of 1–2 and 3–6 intervening genes to indicate different levels of conservation after experimenting with different values as we considered these to be the most clear and informative.

## Abbreviations

HGT: Horizontal gene transfer, EPV: Entomopox viruses, ChPV: Chordopox viruses, OPV: Orthopox viruses, REV: Reticuloendotheliosis virus, EF: enhancing factor, MCL: Markov Cluster Algorithm, NJ: neighbor-joining, ML: maximum-likelihood, MP: maximum-parsimony, IL-10: Interleukin-10, TK: Thymidine Kinase, RRL: Ribonucleotide Reductase large subunit, RRS: Ribonucleotide Reductase small subunit, DP: Deoxyribodipyrimidine Photolyase, HTgene: Horizontally transferred gene, OTU: Operational taxonomic unit

## Authors' contributions

KAB carried out analyses. AMcL devised and supervised the project. KAB and AMcL wrote the paper. Both authors have read and approved the final manuscript.

## Supplementary Material

Additional file 1 **Conserved Poxvirus Synteny**. Horizontal lines represent completely sequenced genomes with genes on the 5'-3' strand and 3'-5' strand represented as blocks above and below the line, respectively. Vertical lines connect genes in different poxviruses that belong to single-copy families conserved in all poxviruses (red lines), all chordopoxviruses (blue lines) and the genus in question (yellow lines). One major inversion between avipox and other chordopox is noticeable, as well as excessive rearrangements in the entomopox lineage, where synteny breaks down. In all other places, synteny is extremely well conserved between genomes. Click here for file

Additional file 2 **Interleukin-10 Phylogeny**. Phylogenetic trees for poxvirus Interleukin-10 and representative non-pox homologs. (A) NJ tree based on amino acid alignment (B) ML tree based on amino acid alignment. Branch lengths are not to scale. (C) ML tree based on nucleotide alignment (gaps copied from amino acid alignment). Branch lengths are not to scale. Tip labels indicate GenBank GI and the common species name for each sequence. Numbers on branches are bootstrap values out of 1000 for NJ tree (A) and out of 100 for ML trees (B and C). Click here for file

Additional file 3 **IL10 multiple sequence alignment**. Each sequence is labelled with its NCBI GI. This image was created using JalView: Clamp, M., Cuff, J., Searle, S. M. and Barton, G. J. (2004), "The Jalview Java Alignment Editor," Bioinformatics, 20, 426-7 Click here for file

Additional file 4 **Synteny conservation around TK in poxvirus genomes**. As for Fig. 2. The gene of interest, TK, is colored red with a bold outline. (A) Neighborhood with respect to "core families"; (B) Neighborhood with respect to "ortholog families"; (C) Neighborhood with respect to "ortho-para families". Click here for file

Additional file 5 **Synteny conservation around RRS in poxvirus genomes**. As for Fig. 2. The gene of interest, RRS, is colored red with a bold outline. Neighborhood with respect to core families. Click here for file

Additional file 6 **Synteny conservation around GP in poxvirus genomes**. As for Fig. 2. The gene of interest, GP, is colored red with a bold outline. Neighborhood with respect to ortholog families. Click here for file

Additional file 7 **Synteny conservation around DP in poxvirus genomes**. As for Fig. 2. The gene of interest, DP, is colored red with a bold outline. Neighborhood with respect to core families. Click here for file

Additional file 8 Multiple sequence alignments for all poxvirus families. Click here for file
