# Supplementary figures and images for: Identification of multiple independent horizontal gene transfers into poxviruses using a comparative genomics approach
Source: BMC Evol Biol. 2008 Feb 27;8:67. doi: 10.1186/1471-2148-8-67 (PMC2268676; doi:10.1186/1471-2148-8-67)

Bratke & McLysaght - Supplementary Figure 1

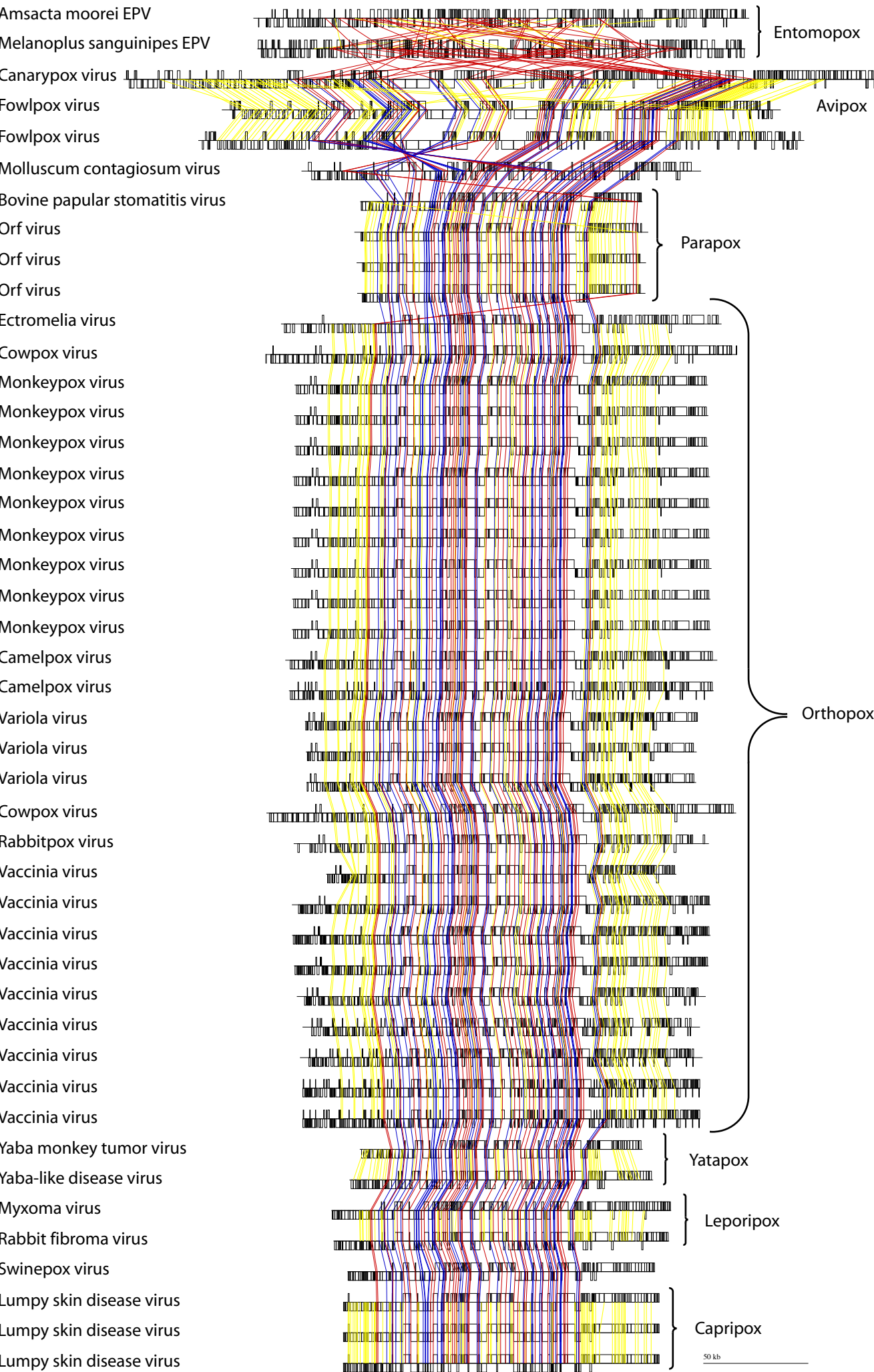

Supplement: Additional file 1 — Conserved Poxvirus Synteny. Horizontal lines represent completely sequenced genomes with genes on the 5'-3' strand and 3'-5' strand represented as blocks above and below the line, respectively. Vertical lines connect genes in different poxviruses that belong to single-copy families conserved in all poxviruses (red lines), all chordopoxviruses (blue lines) and the genus in question (yellow lines). One major inversion between avipox and other chordopox is noticeable, as well as excessive rearrangements in the entomopox lineage, where synteny breaks down. In all other places, synteny is extremely well conserved between genomes. [file 1471-2148-8-67-S1.pdf]

A

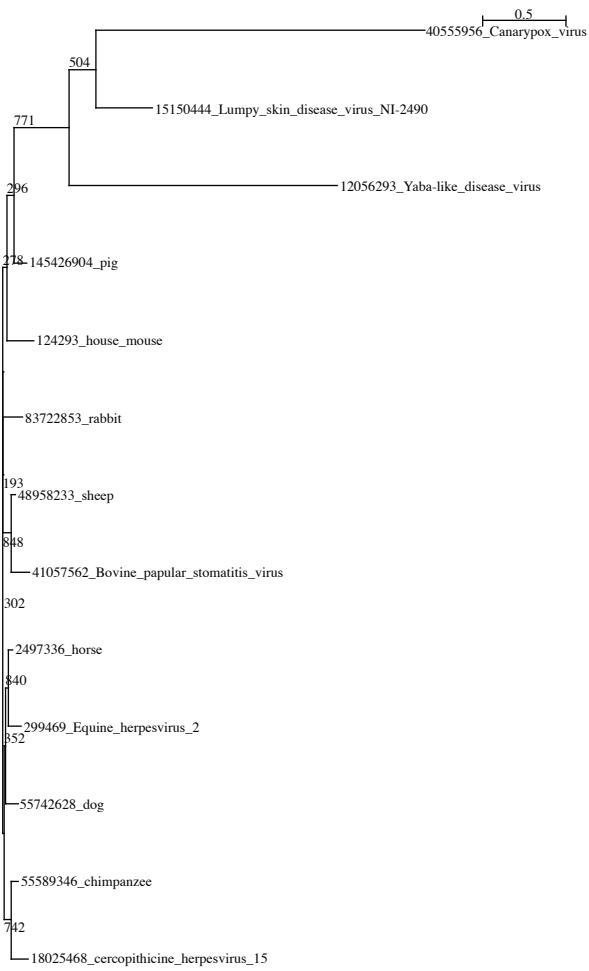

B

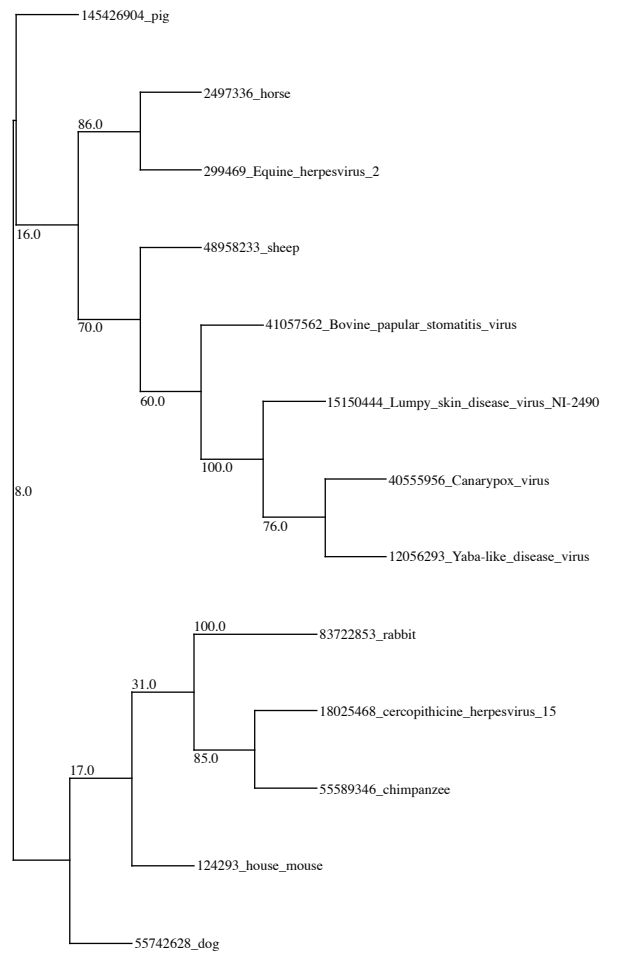

C

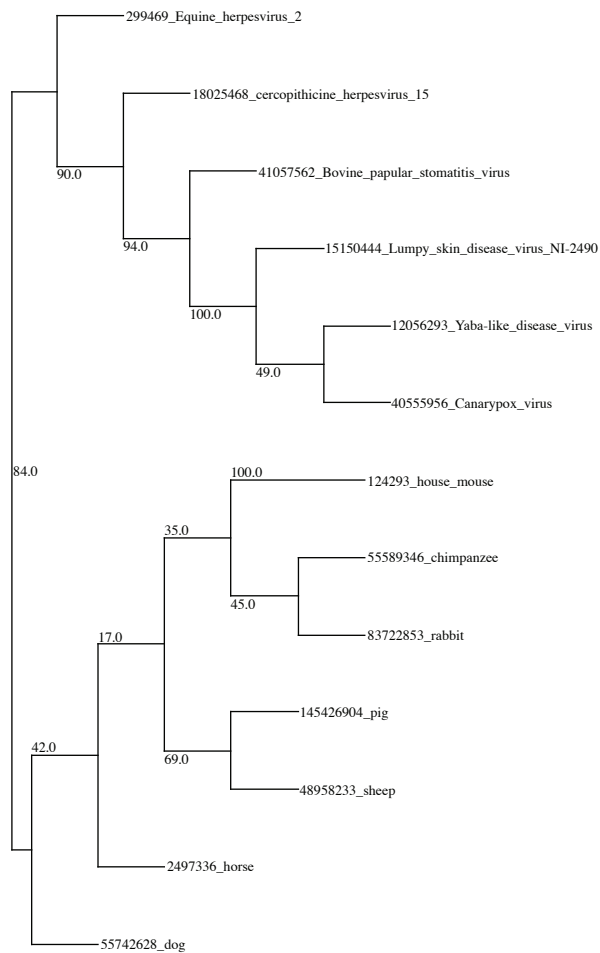

Supplement: Additional file 2 — Interleukin-10 Phylogeny. Phylogenetic trees for poxvirus Interleukin-10 and representative non-pox homologs. (A) NJ tree based on amino acid alignment (B) ML tree based on amino acid alignment. Branch lengths are not to scale. (C) ML tree based on nucleotide alignment (gaps copied from amino acid alignment). Branch lengths are not to scale. Tip labels indicate GenBank GI and the common species name for each sequence. Numbers on branches are bootstrap values out of 1000 for NJ tree (A) and out of 100 for ML trees (B and C). [file 1471-2148-8-67-S2.pdf]

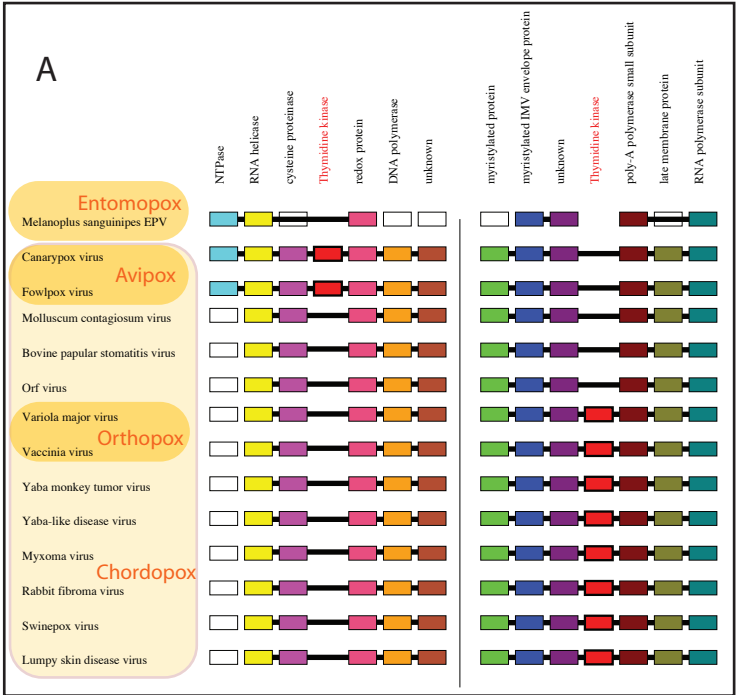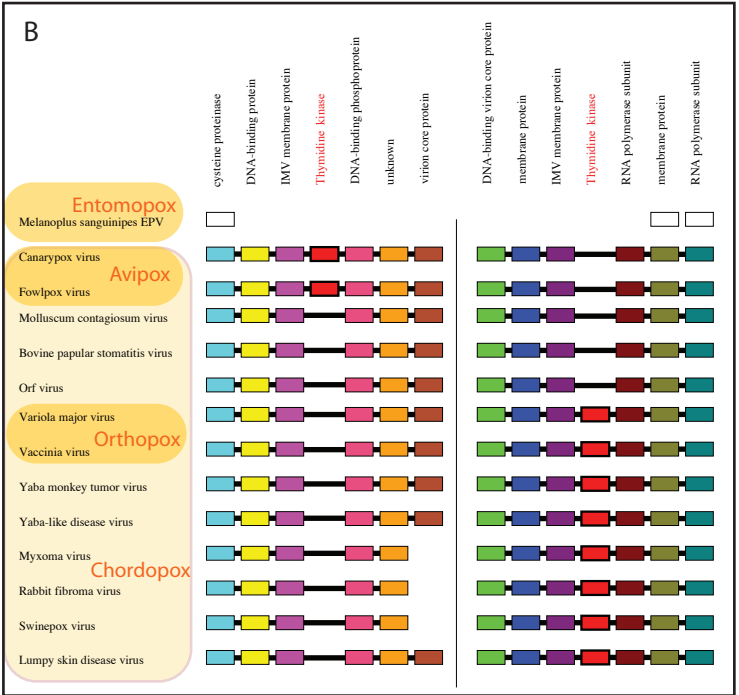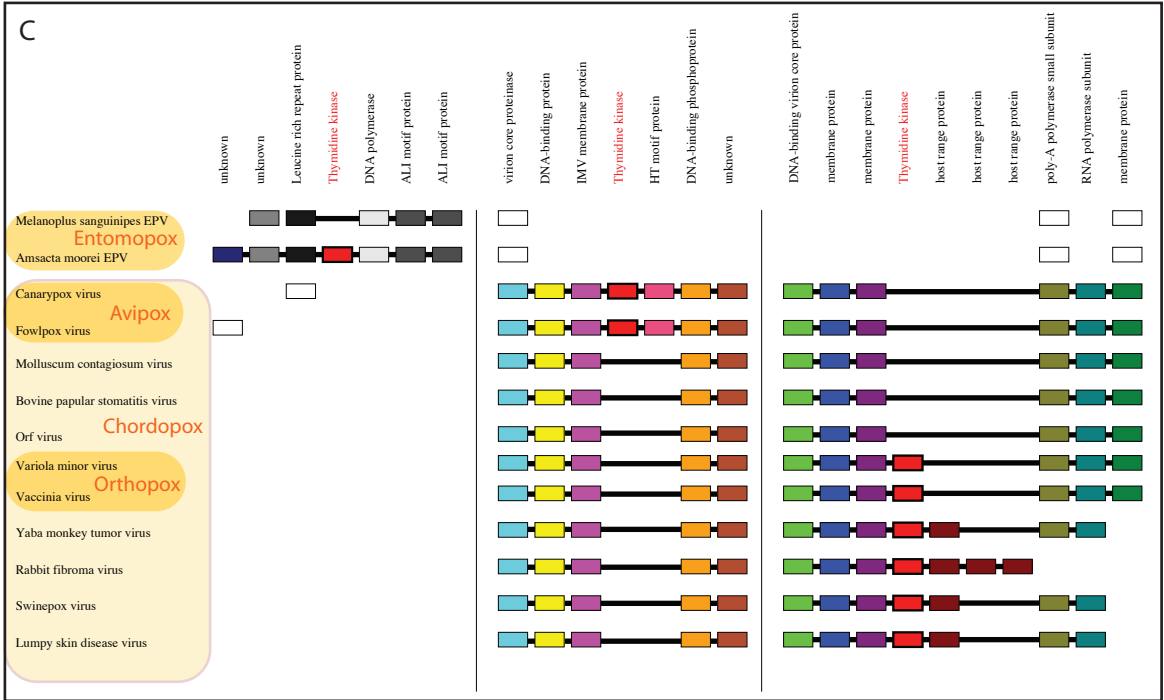

Supplement: Additional file 4 — Synteny conservation around TK in poxvirus genomes. As for Fig. 2. The gene of interest, TK, is colored red with a bold outline. (A) Neighborhood with respect to "core families"; (B) Neighborhood with respect to "ortholog families"; (C) Neighborhood with respect to "ortho-para families". [file 1471-2148-8-67-S4.pdf]

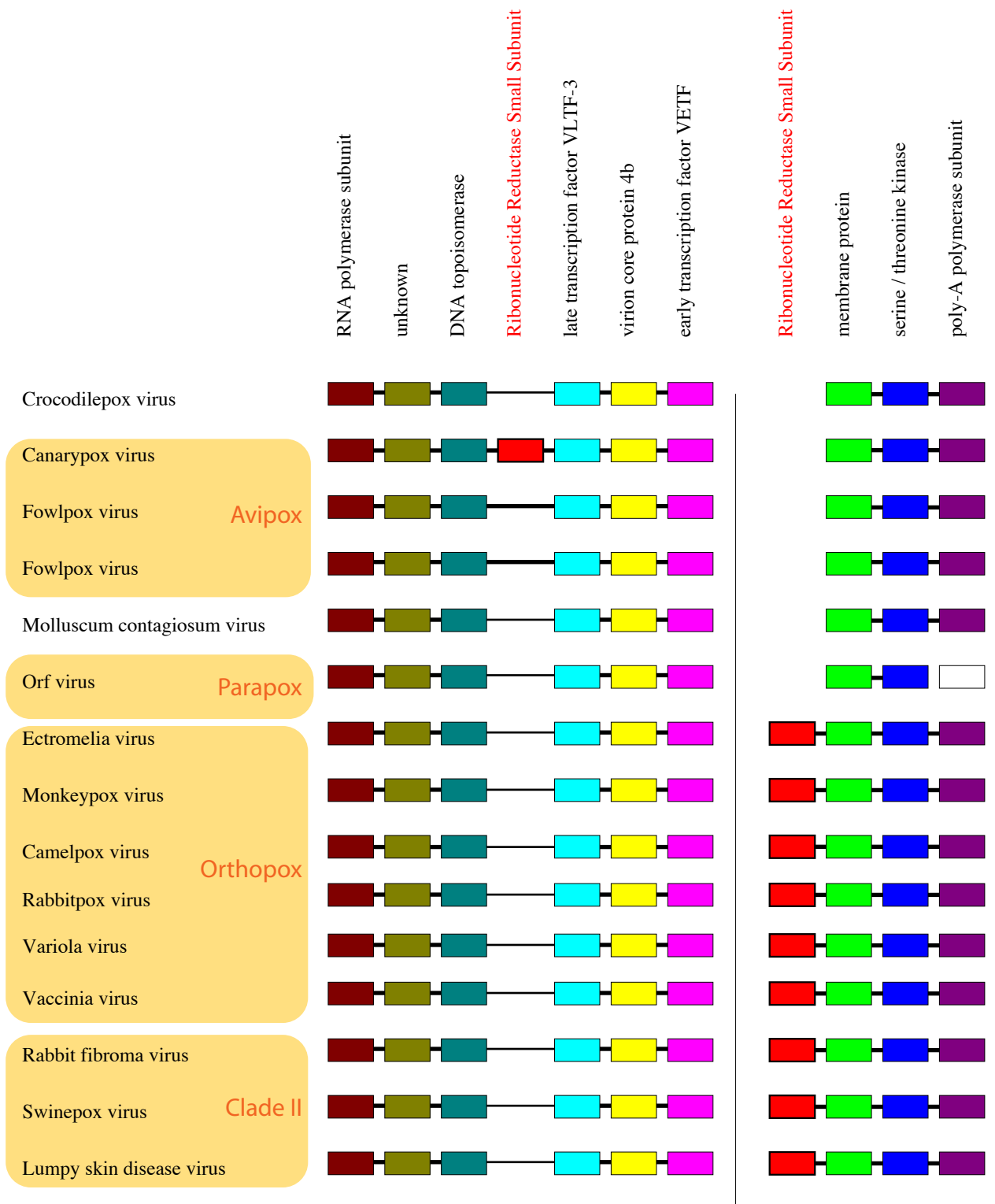

Supplement: Additional file 5 — Synteny conservation around RRS in poxvirus genomes. As for Fig. 2. The gene of interest, RRS, is colored red with a bold outline. Neighborhood with respect to core families. [file 1471-2148-8-67-S5.pdf]

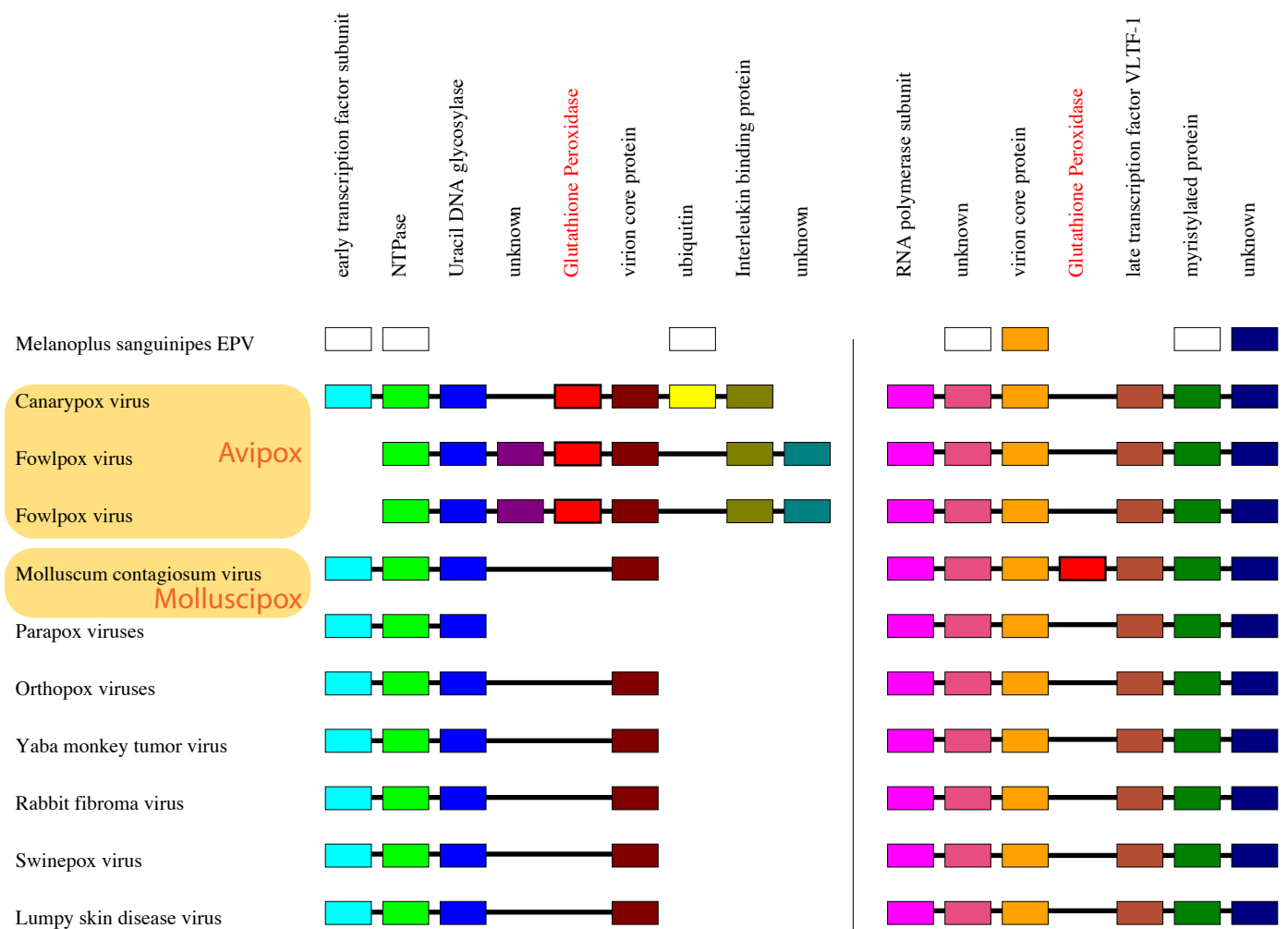

Supplement: Additional file 6 — Synteny conservation around GP in poxvirus genomes. As for Fig. 2. The gene of interest, GP, is colored red with a bold outline. Neighborhood with respect to ortholog families. [file 1471-2148-8-67-S6.pdf]

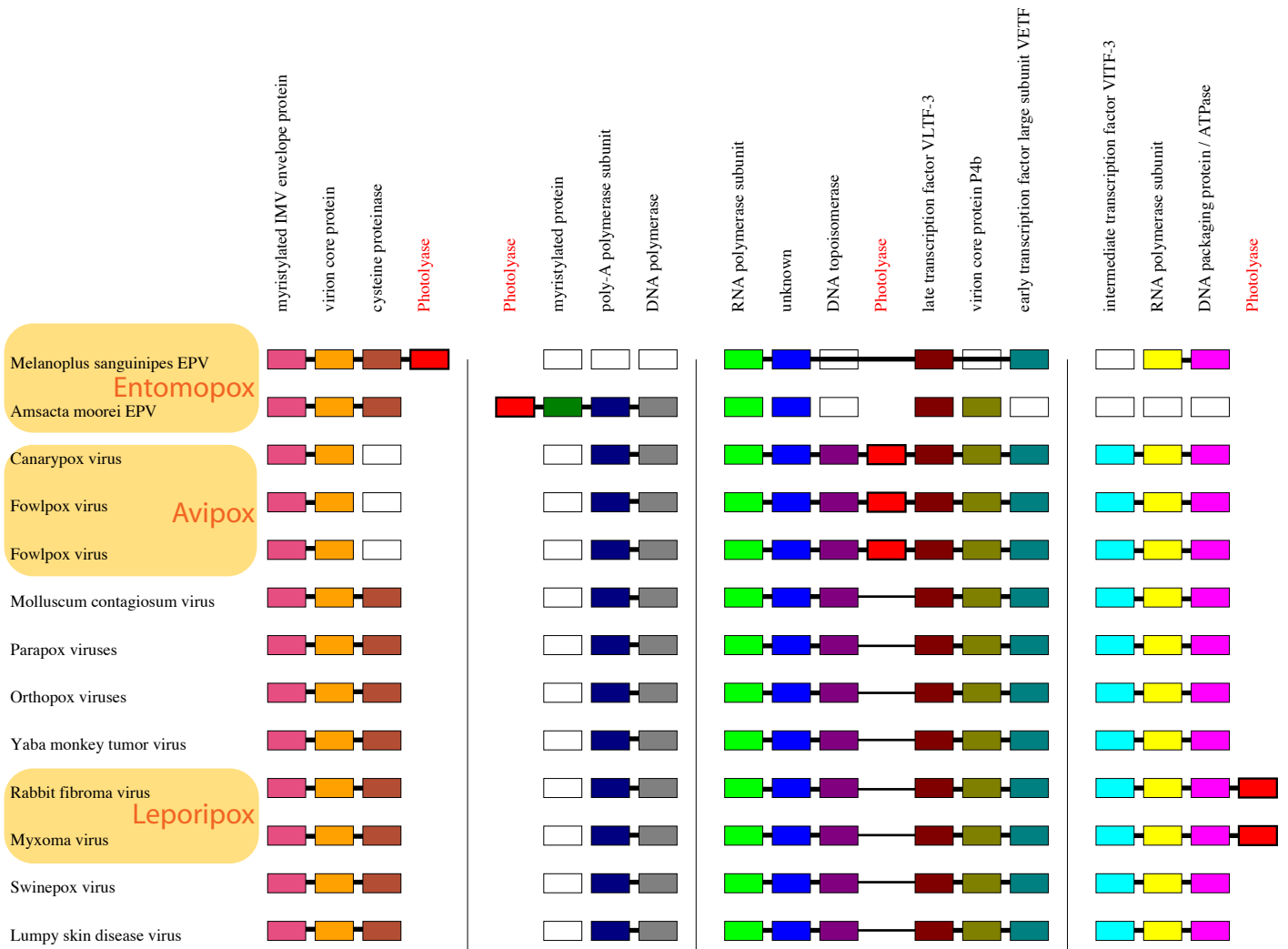

Supplement: Additional file 7 — Synteny conservation around DP in poxvirus genomes. As for Fig. 2. The gene of interest, DP, is colored red with a bold outline. Neighborhood with respect to core families. [file 1471-2148-8-67-S7.pdf]
