# Supplementary material for: Identification of multiple independent horizontal gene transfers into poxviruses using a comparative genomics approach
Source: BMC Evol Biol. 2008 Feb 27;8:67. doi: 10.1186/1471-2148-8-67 (PMC2268676; doi:10.1186/1471-2148-8-67)
Supplement: Additional file 3 — IL10 multiple sequence alignment. Each sequence is labelled with its NCBI GI. This image was created using JalView: Clamp, M., Cuff, J., Searle, S. M. and Barton, G. J. (2004), "The Jalview Java Alignment Editor," Bioinformatics, 20, 426-7 [file 1471-2148-8-67-S3.pdf]

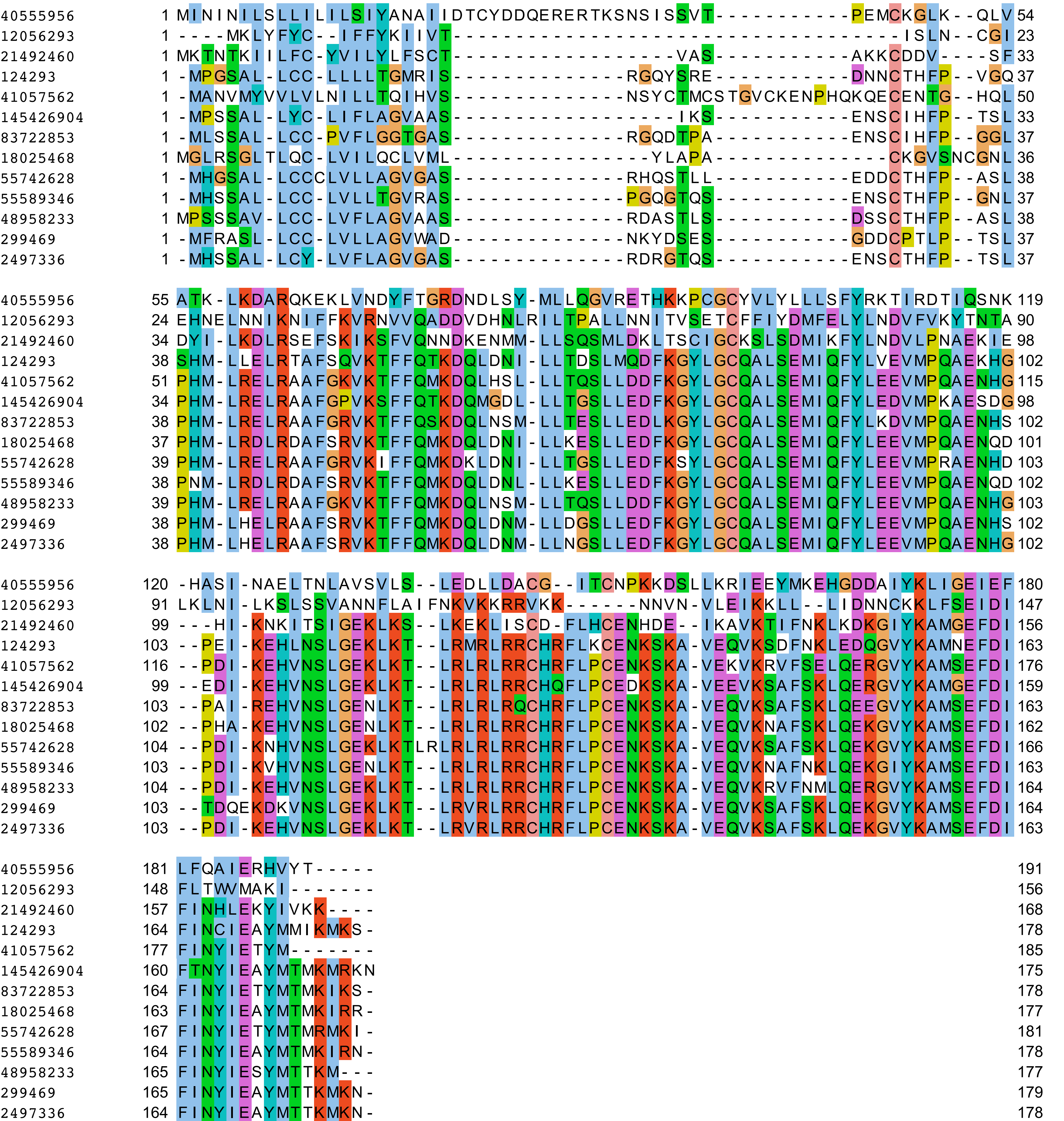
40555956 1 MININILSLLILILSIYANAIIDTCYDDQERERTKSNSISSVT-----------PEMCKGLK--QLV 54
12056293 1 ----MKLYFYC--IFFYKIIVT------------------------------------ISLN--CGI 23
21492460 1 MKTNTKIILFC-YVILYLFSCT------------------VAS-----------AKKCDDV----SF 33
124293 1 -MPGSAL-LCC-LLLLTGMRIS--------------RGQYSRE-----------DNNCTHFP--VGQ 37
41057562 1 -MANVMYVVLVLNILLTQIHVS--------------NSYCTMCSTGVCKENPHQKQECENTG--HQL 50
145426904 1 -MPSSAL-LYC-LIFLAGVAAS------------------IKS-----------ENSCIHFP--TSL 33
83722853 1 -MLSSAL-LCC-PVFLGGTGAS--------------RGQDTPA-----------ENSCIHFP--GGL 37
18025468 1 MGLRSGLTLQC-LVILQCLVML----------------YLAPA--------------CKGVSNCGNL 36
55742628 1 -MHGSAL-LCCCLVLLAGVGAS--------------RHQSTLL-----------EDDCTHFP--ASL 38
55589346 1 -MHSSAL-LCC-LVLLTGVRAS--------------PGQGTQS-----------ENSCTHFP--GNL 37
48958233 1 MPSSSAV-LCC-LVFLAGVAAS--------------RDASTLS-----------DSSCTHFP--ASL 38
299469 1 -MFRASL-LCC-LVLLAGVWAD--------------NKYDSES-----------GDDCPTLP--TSL 37
2497336 1 -MHSSAL-LCY-LVFLAGVGAS--------------RDRGTQS-----------ENSCTHFP--TSL 37
40555956 55 ATK-LKDARQKEKLVNDYFTGRDNDLSY-MLLQGVRETHKKPCGCYVLYLLLSFYRKTIRDTIQSNK 119
12056293 24 EHNELNNIKNIFFKVRNVVQADDVDHNLRILTPALLNNITVSETCFFIYDMFELYLNDVFVKYTNTA 90
21492460 34 DYI-LKDLRSEFSKIKSFVQNNDKENMM-LLSQSMLDKLTSCIGCKSLSDMIKFYLNDVLPNAEKIE 98
124293 38 SHM-LLELRTAFSQVKTFFQTKDQLDNI-LLTDSLMQDFKGYLGCQALSEMIQFYLVEVMPQAEKHG 102
41057562 51 PHM-LRELRAAFGKVKTFFQMKDQLHSL-LLTQSLLDDFKGYLGCQALSEMIQFYLEEVMPQAENHG 115
145426904 34 PHM-LRELRAAFGPVKSFFQTKDQMGDL-LLTGSLLEDFKGYLGCQALSEMIQFYLEDVMPKAESDG 98
83722853 38 PHM-LRELRAAFGRVKTFFQSKDQLNSM-LLTESLLEDLKGYLGCQALSEMIQFYLKDVMPQAENHS 102
18025468 37 PHM-LRDLRDAFSRVKTFFQMKDQLDNI-LLKESLLEDFKGYLGCQALSEMIQFYLEEVMPQAENQD 101
55742628 39 PHM-LRELRAAFGRVKIFFQMKDKLDNI-LLTGSLLEDFKSYLGCQALSEMIQFYLEEVMPRAENHD 103
55589346 38 PNM-LRDLRDAFSRVKTFFQMKDQLDNL-LLKESLLEDFKGYLGCQALSEMIQFYLEEVMPQAENQD 102
48958233 39 PHM-LRELRAAFGKVKTFFQMKDQLNSM-LLTQSLLDDFKGYLGCQALSEMIQFYLEEVMPQAENHG 103
299469 38 PHM-LHELRAAFSRVKTFFQMKDQLDNM-LLDGSLLEDFKGYLGCQALSEMIQFYLEEVMPQAENHS 102
2497336 38 PHM-LHELRAAFSRVKTFFQMKDQLDNM-LLNGSLLEDFKGYLGCQALSEMIQFYLEEVMPQAENHG 102
40555956 120 -HASI-NAELTNLAVSVLS--LEDLLDACG--ITCNPKKDSLLKRIEEYMKEHGDDAIYKLIGEIEF 180
12056293 91 LKLNI-LKSLSSVANNFLAIFNKVKKRRVKK------NNVN-VLEIKKLL--LIDNNCKKLFSEIDI 147
21492460 99 ---HI-KNKITSIGEKLKS--LKEKLISCD-FLHCENHDE--IKAVKTIFNKLKDKGIYKAMGEFDI 156
124293 103 --PEI-KEHLNSLGEKLKT--LRMRLRRCHRFLKCENKSKA-VEQVKSDFNKLEDQGVYKAMNEFDI 163
41057562 116 --PDI-KEHVNSLGEKLKT--LRLRLRRCHRFLPCENKSKA-VEKVKRVFSELQERGVYKAMSEFDI 176
145426904 99 --EDI-KEHVNSLGEKLKT--LRLRLRRCHQFLPCEDKSKA-VEEVKSAFSKLQERGVYKAMGEFDI 159
83722853 103 --PAI-REHVNSLGENLKT--LRLRLRQCHRFLPCENKSKA-VEQVKSAFSKLQEEGVYKAMSEFDI 163
18025468 102 --PHA-KEHVNSLGENLKT--LRLRLRRCHRFLPCENKSKA-VEQVKNAFSKLQEKGVYKAMSEFDI 162
55742628 104 --PDI-KNHVNSLGEKLKTLRLRLRLRRCHRFLPCENKSKA-VEQVKSAFSKLQEKGVYKAMSEFDI 166
55589346 103 --PDI-KVHVNSLGENLKT--LRLRLRRCHRFLPCENKSKA-VEQVKNAFNKLQEKGIYKAMSEFDI 163
48958233 104 --PDI-KEHVNSLGEKLKT--LRLRLRRCHRFLPCENKSKA-VEQVKRVFNMLQERGVYKAMSEFDI 164
299469 103 --TDQEKDKVNSLGEKLKT--LRVRLRRCHRFLPCENKSKA-VEQVKSAFSKLQEKGVYKAMSEFDI 164
2497336 103 --PDI-KEHVNSLGEKLKT--LRVRLRRCHRFLPCENKSKA-VEQVKSAFSKLQEKGVYKAMSEFDI 163
40555956 181 LFQAIERHVYT----- 191
12056293 148 FLTWWMAKI------- 156
21492460 157 FINHLEKYIVKK---- 168
124293 164 FINCIEAYMMIKMKS- 178
41057562 177 FINYIETYM------- 185
145426904 160 FTNYIEAYMTMKMRKN 175
83722853 164 FINYIETYMTMKIKS- 178
18025468 163 FINYIEAYMTMKIRR- 177
55742628 167 FINYIETYMTMRMKI- 181
55589346 164 FINYIEAYMTMKIRN- 178
48958233 165 FINYIESYMTTKM--- 177
299469 165 FINYIEAYMTTKMKN- 179
2497336 164 FINYIEAYMTTKMKN- 178
